# Valorization of the green waste parts from sweet potato (*Impoea batatas* L.): Nutritional, phytochemical composition, and bioactivity evaluation

**DOI:** 10.1002/fsn3.1675

**Published:** 2020-07-14

**Authors:** Jingyang Hong, Taihua Mu, Hongnan Sun, Aurore Richel, Christophe Blecker

**Affiliations:** ^1^ Laboratory of Food Chemistry and Nutrition Science Institute of Food Science and Technology Chinese Academy of Agricultural Sciences Beijing China; ^2^ Key Laboratory of Agro‐Products Processing Ministry of Agriculture and Rural Affairs Beijing China; ^3^ Biological and Industrial Chemistry Unit Gembloux Agro‐Bio Tech University of Liège Gembloux Belgium; ^4^ Department of Food Science and Formulation Gembloux Agro‐Bio Tech University of Liège Gembloux Belgium

**Keywords:** antioxidant activity, chemical/nutritional characterization, correlation analysis, gray relational analysis, sunscreen activity, sweet potato leaves

## Abstract

In the present study, leaves from 13 sweet potato cultivars were collected as raw materials. The nutritional and functional composition, antioxidant activity, and sunscreen activity of different sweet potato leaf samples were determined, and the comprehensive nutritional quality was calculated by gray relational analysis. Results showed that the nutritional and functional components are significantly different between different cultivars. Tainong71 showed the highest comprehensive nutritional quality, followed by Fu22, Ningcai, Fu23, Ecai10, Zhecai726, Ecai1, Fu18, Pushu53, Guangcai5, Shulv1, Guangcai2, and Zhecai1. The antioxidant activity varied from 3.94 to 16.75 g Trolox equivalent/100 g dry weight. Pushu53 showed the highest sunscreen activity, with the sun protection factor 24.65. There was a positive correlation between antioxidant activity and sunscreen activity (*r* = .737, *p* = .004). In conclusion, sweet potato leaves possess high nutritional and functional properties, and have the huge potential to be used as green leafy vegetables and sunscreen agent.

## Graphical abstract

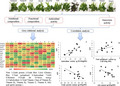

## 1 INTRODUCTION

Sweet potato (*Impoea batatas* L.) is one of the most important food crops and widely grown around the world (de Albuquerque, Sampaio, & de Souza, 2019). Sweet potato leaves are the above‐ground part of sweet potato, which can be harvested 3–4 times in 1 year. The annual yield of sweet potato leaves is almost the same with root. Sweet potato leaves have become a new kind of vegetable in the United States, Japan, Taiwan, and Hong Kong. But in most areas of China, sweet potato leaves are still discarded as waste directly, resulting in huge waste of resources and the pollution of environment (Lu, Zhou, Ren, & Zhang, 2019). In recent years, there are increasing studies concentrated on the sweet potato leaves. Islam (2006) reported that sweet potato leaves have positive effects on human health and nutrition. Sun, Mu, Xi, Zhang, and Chen (2014) studied the nutritional compositions of leaves from 40 sweet potato cultivars and found that sweet potato leaves, which contain several nutrients and bioactive compounds, should be consumed as leafy vegetables in an attempt to reduce malnutrition. Although thousands of sweet potato leaf cultivars have been reported, information about nutrition and function of sweet potato leaves is still deficient.

Nutritional components are the main indicators for evaluating the nutritional value of sweet potato leaves. At present, judging the nutritional value of food from single component is inaccurate and incomprehensive. The gray relational analysis (GRA) is a technique of system theory that is used to evaluate the comprehensive nutritional value. Nowadays, GRA has been applied to evaluate the nutritional quality of different crops and the ideal varieties have been successfully selected (Liu et al., 2017). So it is sensible to choose GRA to evaluate the comprehensive nutritional value of different varieties of sweet potato leaves in this study.

In addition, ultraviolet radiation (UV) is the main cause of most skin diseases, especially skin cancer. The incidence of skin cancer induced by ultraviolet radiation has risen sharply all over the world. Chemical protection is one of the important ways to protect skin from UV, but long‐term use of chemicals will change the active state of macrophages and break the immune balance of the body (Rubio, Valverde‐Som, Sarabia, & Ortiz, 2019). In clinic, the main anti‐radiation drugs are ammonia‐mercapto, which can cause nausea, vomiting, hypertension, and other adverse reactions (Clémenson et al., 2019). So it is urgent to develop natural materials to protect skin from UV radiation. Studies have shown that both oral and topical application of polyphenols can significantly prevent skin from damage and skin cancer, such as green tea, pomegranate, and mulberry (Afaq & Katiyar, 2012; Hu, Zhang, Chen, & Wang, 2017). UV can form reactive oxygen species (ROS) which might react with oxygen molecules in human cells and prevent the body destruction by oxidative reactions (Ho et al., 2007). However, there is no relevant report on the prevention of UV by polyphenols from sweet potato leaves.

Therefore, in the present study, sweet potato leaves from 13 sweet potato cultivars were collected, and the nutritional and functional components, antioxidant activity, and sunscreen activity were determined. The comprehensive nutritional value was evaluated by GRA, so as to provide some theoretical support for the effective development and utilization of sweet potato leaves.

## 2 MATERIAL AND METHODS

### 2.1 Materials

Sweet potato leaves from 13 sweet potato cultivars (Guangcai2, Guangcai5, Ecai1, Ecai10, Zhecai1, Zhecai726, Fu18, Fu22, Fu23, Tainong71, Shulv1, Pushu53, Ningcai) were obtained from Agricultural Machinery Extension Station in Beijing, China. They were cleaned with tap water and lyophilized in freeze dryer machine (FD5‐3, SIM USA Intl. Group) at −57°C for 96 hr and then ground into powder by an ultrafine grinder. Powdered samples were stored in well‐labeled aluminum foil bag at −4°C until analyzed.

### 2.2 Proximate compositions

Ash, crude fat, and crude protein contents were determined by AOAC methods (AOAC 923.03, 960.39, and 976.05, respectively). Crude fiber (g/100 g DW) was determined by ISO method 5498:1981. Carbohydrate content (g/100 g DW) was calculated by subtracting the sum of ash, crude fat, crude protein, and crude fiber contents from 100. Gross energy (kcal/100 g DW) was calculated according to the European Universal Energy Coefficient (Menezes et al., 2016), with the following Equation

(1) $${\text{ME}}_{\text{food}}=4\times P+9\times F+4\times \text{AC}$$

ME_food_: metabolizable energy of food (kcal/100 g); P: protein content (g/100 g); F: crude fat content (g/100 g); AC: carbohydrate content (g/100 g).

### 2.3 Mineral content

Leaf samples were digested in concentrated HNO_3_ (AOAC, 2000). The digest was transferred to a 25 ml volumetric flask, and the volume was adjusted to 25 ml with deionized water. A blank digest was prepared in a similar manner. Mineral content, expressed as mg mineral/100 g DW, was determined by inductively coupled plasma atomic emission spectrometry (ICAP6000, Thermo Fisher Scientific).

### 2.4 Vitamin content

Vitamin C (VC), vitamin E (VE),vitamin B_1_ (VB_1_), vitamin B_2_ (VB_2_), vitamin B_3_ (VB_3_), and folic acid were extracted and determined by a slightly modified HPLC method previously reported by Gratacós‐Cubarsí, Sárraga, Clariana, Regueiro, and Castellari (2011). Briefly, 1 g of sample was mixed with 9 ml of 0.1 M hydrochloric acid and maintained at 100°C for 30 min in a water bath. After cooling, 6 ml of 2.5 M sodium acetate and 1 ml of 10% (w/v) taka‐diastase solution were added. Samples were incubated overnight at 37°C and centrifuged at 500 *g* for 5 min at 4°C. The resulting supernatant was adjusted to 20 ml with ultrapure water. An aliquot (5 ml) was purified using an Oasis MCX cartridge (6cc‐150 mg, Waters Corp.) for the simultaneous determination of vitamins C, E, B_1_, B_2_, B_3_, and folic acid.

β‐carotene was determined via the slightly modified protocol of Kourouma, Mu, Zhang, and Sun (2019), and 2 g of sweet potato leaves powder was mixed with 20 ml petroleum ether: acetone (80:20, v/v) for 20 min at 40°C on ultrasonic water bath under dim light for carotenoids extraction. The extraction was repeated three times. The extracts were collected after centrifuge 10 min at 7,000 *g* and concentrated under rotary vacuum evaporator at 30°C to get 4 ml of final extract. Every 1 ml of extract was dried under nitrogen gas, re‐dissolved in 1 ml petroleum ether, filtered through 0.45 μm, and analyzed by HPLC.

Quantification of carotenoids was performed using reversed‐phase high‐performance liquid chromatography (RP‐HPLC, Shimadzu LC‐20A) on column C_18_ (150 mm × 4.6 mm; 5 μm particle size) with mobile phase of methanol‐acetonitrile (90:10, v/v) at flow rate of 1 ml/min at 25°C. The injection volume was 20 μl, and the detection wavelength was 450 nm.

### 2.5 Amino acid composition

The amino acid composition of leaf sample was obtained using the Biochrom 3.1 amino acid analyzer according to the method by Bártová, Bárta, Brabcová, Zdráhal, and Horáčková (2015) with appropriate modifications. Briefly, 10 ml of 6 N hydrochloric acid was added to 100 mg sample in test tube. Blow the sample with nitrogen for 1 min, then covered and hydrolyzed in an oven at 110°C for 24 hr, and allowed to cool to room temperature. The hydrolysate was filtered to remove visible sediments and evaporated to dryness under vacuum at 60°C. The hydrolysate was dissolved in 1 ml of 0.02 N hydrochloric acid. An aliquot (20 μl) was injected into the amino acid analyzer (tryptophan could not be determined by this method). The amino acid score (AAS) was calculated with reference to FAO/WHO (Joint WHO/FAO/UNU Expert Consultation, 2007) reference amino acid pattern (Esan, Omoba, & Enujiugha, 2018).

(2) $$\text{AAS}=\left(\text{limiting}\;\text{amino}\;\text{acid}\right)/\left(\text{Reference}\;\text{amino}\;\text{acid}\right)\times 100$$

The reference levels of each EAA (mg/g protein) were as follows: lysine, 45; histidine, 15; threonine, 23; valine, 39; isoleucine, 30; leucine, 59; methionine and cystine, 16; phenylalanine and tyrosine, 30.

### 2.6 Total polyphenol content (TPC) and antioxidant activity

Total polyphenol content was measured by the Folin–Ciocalteu method with a slight modification (Figueiredo et al., 2014). Polyphenols were extracted according to the method of Sun et al. (2014). A calibration curve was generated with chlorogenic acid standards (Sigma‐Aldrich, Inc.), ranging from 0.02 to 0.10 mg/ml. The linear regression equation was

(3) y=0.8761x+0.0068

and *R* ^2^ = .9994. TPC was expressed as milligram chlorogenic acid equivalents (CAE) per gram leaf powder on a DW basis. TPC was calculated according to the following equation:

(4) $$\text{TPC}=\left(A-0.0068\right)/8.7671\times V/M$$

where *A* is the absorbance, *V* is the volume of the crude extract diluent (ml), and *M* is the mass of the tested sample on a DW basis (g).

Antioxidant activity of the leaf samples was determined with the Ferric ion reducing antioxidant power (FRAP) (Goel, Irshad, Mehdi, Rizvi, & Ahmad, 2013). FRAP values were expressed as grams Trolox equivalents (TE) per 100 g leaf powder on a DW basis.

### 2.7 SPF

One gram of each sample was diluted with 20 ml ethanol and extracted by ultrasonic method for 30 min and centrifuge at 7,500 *g* for 10 min, repeated for three times; collect centrifugal fluid, constant volume to 100 ml. After preparation, all the samples were scanned at wavelength between 290 and 320 nm, in the range of UVB, every 5 nm, and three replicates were made at each point. In the end of all measurements, the Mansur equation was applied to calculate SPF values (Prakash, Lokesh, & Manral, 2015).

(5) $$\text{SPF}=\text{CF}\times \sum_{290}^{320}\text{EE}\left(\lambda \right)\times I\left(\lambda \right)\times \text{Abs}\left(\lambda \right)$$

Here, CF = correction factor (10), EE (*λ*) = erythmogenic effect of radiation with wavelength *λ*, Abs (*λ*) = spectro‐photometric absorbance values at wavelength *λ*. The values of EE (*λ*) × *I* are constants. They were determined by Sayre, Agin, LeVee, & Marlowe, 1979. The values of EE (*λ*) × *I* from 290–320 nm were 0.0150, 0.0817, 0.2874, 0.3278, 0.1864, 0.0837, 0.0180, respectively.

### 2.8 Comprehensive nutritional value

In this study, the leaf samples represent a gray system; each cultivar is a factor in the system. The nutritional value correlation between the samples and an ideal sample was determined. Based on the aim of this study, the ideal sample was selected by combining the upper or lower nutritional contents. Crude protein, dietary fiber, mineral content, vitamins, total polyphenol content, antioxidant activity, etc., which are positively correlated with nutritional content, utilized 5% of the maximum value of the tested leaves. However, crude fat, carbohydrate, gross energy, etc., which are negatively correlated with the nutritional content, utilized 5% of the minimum value of the tested leaves. A high correlation coefficient is indicative that the degree of similarity between the sample and the ideal sample is high. The correlation coefficient was calculated according to the method reported by Kadier (Kadier et al., 2015). Assuming that the ideal list was *X*_0_, the compared list was *Xi*, *i* = 1,2,3… …, and *X*_0_ = {*X*_0_(1), *X*_0_(2), *X*_0_(3) … …*X*_0_(*k*)}, *Xi* = {*Xi*(1), *Xi*(2), *Xi*(3)… …*Xi*(*k*)}, *k* = 1,2,3… …*M*. The correlation coefficient between the samples and ideal sample at the *k* point was calculated using the following equation:

(6) $$\zeta_{i(k)}=\frac{\min\min\left|\Delta i\left(k\right)\right|+\rho \max\max\left|\Delta i\left(k\right)\right|}{\left|\Delta i\left(k\right)\right|+\rho \max\max\left|\Delta i\left(k\right)\right|}$$

where Δ*i*(*k*) = |*X*_0_(*k*) − *Xi*(*k*)|, min|Δ*i*(*k*)| is the minimum value of the first level, min min|*Δ*i(*k*)| is the minimum value of the second level, max|Δ*i*(*k*)| is the maximum value of the first level, and max max|Δ*i*(*k*)| is the maximum value of the second level. In Equation 6, *ρ* (0 ≤ *ρ* ≤ 1) is the distinguishing coefficient. The distinguishability was increased with the *ρ* value decreased. In this study, *ρ* was set to .5, because this value offers moderate distinguishing effects and good stability. The average gray relational coefficient at the *k* point was determined using the following equation:

(7) $$\gamma_{k}=\frac{1}{N}\sum_{i=1}^{n}\zeta_{i\left(k\right)}$$

The weight at the *k* point was calculated with the following equation:

(8) $$W_{k}=\frac{\gamma_{k}}{\sum_{1}^{M}\gamma_{k}}$$

The gray relational degree was determined by the following equation:

(9) $$G_{i}=\sum_{k=1}^{M}\zeta_{i\left(k\right)}W_{k}$$

### 2.9 Statistical analysis

All the experiments were carried out in triplicate. Statistical analyses were performed using the Statistical Product and Service Solutions software (IBM SPSS Statistical 21). Statistical significance was set to *p* < .05.

## 3 RESULTS AND DISCUSSION

### 3.1 Nutritional and functional composition

Table 1 shows the proximate compositions of leaves from 13 sweet potato cultivars. The moisture content ranged between 87.37 and 90.27 g/100 g FW. Shulv1 had the highest moisture content (90.27 ± 0.17 g/100 g FW), while Fu22 had the lowest moisture content (87.37 ± 0.82 g/100 g FW). The moisture contents obtained in this study were similar to those reported by Ishida et al. (2000). The moisture content of sweet potato leaves may be affected by the harvest time.

**TABLE 1:** Moisture, crude protein, crude fat, crude fiber, ash, dietary fiber, carbohydrate content and gross energy of leaves from 13 sweet potato cultivars (g/100 g DW)

Cultivar	Moisture^a^	Crude protein	Crude fat	Crude fiber	Ash	Dietary fiber	Carbohydrate	Gross energy^b^
Guang2	89.67 ± 0.87bc	33.64 ± 0.83c	3.87 ± 0.64cd	10.92 ± 0.07f	15.62 ± 0.05f	37.28 ± 0.1a	36.31 ± 0.49d	311.66 ± 2.35ab
Guang5	88.67 ± 1.34abc	31.41 ± 0.69b	2.75 ± 0.41a	9.26 ± 0.03a	14.86 ± 0.05d	38.87 ± 0.33bcd	41.98 ± 0.55fg	316.23 ± 0.32bc
Ecai1	87.92 ± 0.43ab	35.66 ± 0.2de	4.28 ± 0.92d	9.82 ± 0.08bc	13.43 ± 0.15a	40.32 ± 0.1f	36.79 ± 1.24d	329.1 ± 7.34e
Ecai10	89.95 ± 0.16bc	38.52 ± 0.33f	4.25 ± 0.33d	10.63 ± 0.01e	16.61 ± 0.12h	38.71 ± 0.01bcd	30.13 ± 0.74a	312.26 ± 2.41ab
Zhecai1	89.89 ± 0.36bc	35.45 ± 0.31d	2.78 ± 0.23a	9.74 ± 0.12b	15.51 ± 0.03ef	38.48 ± 0.42bc	36.75 ± 0.88d	311.93 ± 1.56ab
Zhe726	90.01 ± 1.2bc	33.65 ± 0.34c	2.74 ± 0.22c	9.91 ± 0.09c	14.61 ± 0.18c	39.06 ± 0.3d	38.15 ± 0.3e	320.25 ± 1.67cd
Fu18	88.41 ± 0.98abc	36.44 ± 0.25e	2.78 ± 0.23ab	10.19 ± 0.02d	16.48 ± 0.03h	38.91 ± 0.04e	34.01 ± 0.19bc	307.93 ± 0.72a
Fu22	87.37 ± 0.82a	28.01 ± 0.19a	2.74 ± 0.22a	10.11 ± 0.02d	16.47 ± 0.01h	41.45 ± 0.11cd	42.64 ± 0.12g	307.62 ± 1.45a
Fu23	87.96 ± 2.03ab	36.16 ± 0de	2.75 ± 0.06a	11.4 ± 0.06g	15.45 ± 0.07e	40.35 ± 0.14g	34.22 ± 0.19bc	306.25 ± 0.08a
Taninong71	88.24 ± 0.13abc	35.49 ± 0.07d	3.3 ± 0.21abc	10.2 ± 0.07d	15.93 ± 0.07g	40.06 ± 0.13f	35 ± 0.34c	312.11 ± 1.35ab
Shulv1	90.27 ± 0.17c	36.04 ± 0.14de	3.03 ± 0.75ab	9.77 ± 0.06bc	16.99 ± 0.1i	39.58 ± 0.14bc	33.64 ± 0.03b	310.56 ± 0.92ab
Pushu53	88.61 ± 0.02abc	31.36 ± 0.2b	3.25 ± 0.08abc	9.39 ± 0a	13.74 ± 0.14b	38.48 ± 0.13f	42.19 ± 0.19fg	323.42 ± 0.63d
Ningcai	88.14 ± 0.4abc	31.14 ± 0.08b	2.49 ± 0.56a	10.66 ± 0.05e	14.88 ± 0.02d	38.42 ± 0.14b	41.11 ± 0.45f	308.89 ± 2.23a

Note

Data are means ± *SD* (*n* ≥ 2). Values within columns with different letters are significantly different (*p* < .05).

Abbreviations: DW, dry weight; FW, fresh weight.

^a^ Moisture content was expressed in g/100 g FW.

^b^ Gross energy was expressed in kcal/100 g DW.

Protein is an essential nutrition in the human diet (Pereira & Vicente, 2013). The direct consumption of vegetable proteins in food products has been increasing over the years because of animal‐related diseases, global shortage of animal protein, increasing demand for wholesome or religious food, and for economic reasons (Asgar, Fazilah, Huda, Bhat, & Karim, 2010). From the Table 1, we can see that protein content ranged from 28.01 to 38.52 g/100 g DW in sweet potato leaves. There was a significant difference in protein content among different cultivars. It was higher than the contents of Japan's two cultivars Kogannesengan (KS) and Beniazuma (BA) which was reported by Ishida et al. (2000). The crude protein content of KS and BA was 29.5 g/100 g DW and 24.5 g/100 g DW, respectively.

Crude fiber content varied from 9.26 to 11.4 g/100 g DW while the dietary fiber content ranged from 37.28 to 41.45 g/100 g DW among different sweet potato cultivars. Sweet potato leaves can be used as a good plant source of dietary fiber. Fu23 has the highest crude fiber content (11.4 ± 0.06 g/100 g DW). It is higher than the crude fiber content of black tea from China (11.29 g/100 g) and India (11.26 g/100 g) (Śmiechowska & Dmowski, 2006). This may be related to the differences of sweet potato leaf varieties, maturity.

The ash content ranged from 13.43 ± 0.15 to 16.99 ± 0.1 g/100 g DW; it was higher than many other vegetables such as radish, garlic, and yam which is reported by Sipahioglu and Barringer (2003). Ash generally represents the total amount of inorganic elements which has important physiological and pathological significance in human life activities. Additionally, carbohydrate and gross energy of sweet potato leaf were 30.13 ± 0.74 to 42.19 ± 0.19 g/100 g DW and 306.25 ± 0.08 to 323.42 ± 0.63 g/100 g DW. The average contents of carbohydrate and gross energy was 37.15 g/100 g DW and 313.71 kcal/100 g.

### 3.2 Mineral content

Table 2 shows the mineral content of leaves from 13 sweet potato cultivars. Minerals are classified into two groups: macroelements (Ca, K, P, Mg, and Na) and microelements (Fe, Mn, Zn, and Cu). In this study, Ca ranged from 1,002.90 (Ningcai) to 1,582.36 (Fu18) mg/100 g DW; K ranged from 5,321.62 (Ecai1) to 7,720.68 (Shulv1) mg/100 g DW; P ranged from 663.79 (Ecai10) to 1,016.02 (Shulv1) mg/100 g DW; Mg ranged from 438.70 (Ningcai) to 761.25 (Zhecai1) mg/100 g DW; and Na ranged from 34.92 (Shulv1) to 197.52 (Fu23) mg/100 g DW.

**TABLE 2:** The contents of minerals (mg/100 g DW), vitamins (mg/100 g DW) and total polyphenols (TPC) (g CAE/100 g DW), and antioxidant activity (g TE/100 gDW) of leaves from 13 sweet potato cultivars

Cultivar	K	Na	Ca	Mg	P	Fe	Mn	Zn	Cu
Guangcai2	5,755.53 ± 454.23ab	36.01 ± 1.91a	1,211.37 ± 114.06abc	546.45 ± 46.18ab	719.54 ± 27abc	26.93 ± 19.94	7.62 ± 2.42	2.41 ± 0.19a	0.68 ± 0.09ab
Guangcai5	5,144.36 ± 481.12a	180.15 ± 12.87e	1,276.66 ± 126.73abc	592.23 ± 49.46bc	701.12 ± 16.88ab	19.16 ± 2.35	8.5 ± 2.99	2.24 ± 0.4a	0.5 ± 0.05a
Ecai1	4,999.18 ± 456a	47.75 ± 6.28abc	954.64 ± 108.7ab	583.41 ± 67.17bc	749.82 ± 40.03abc	10.89 ± 1.59	7.59 ± 2.36	2.39 ± 0.34a	0.82 ± 0.13b
Ecai10	6,431.89 ± 822.35ab	88.43 ± 13.39cd	1,348.56 ± 190.07bc	678.19 ± 98.62bc	626.98 ± 52.06a	18.69 ± 5.11	7.73 ± 2	2.37 ± 0.37a	0.6 ± 0.11ab
Zhecai1	5,417.75 ± 155.14ab	44.61 ± 0.6ab	1,389.12 ± 243.45c	727.41 ± 47.86c	810.95 ± 8.03bc	24.49 ± 1.17	9.98 ± 3.39	2.83 ± 0.01a	0.61 ± 0.06ab
Zhecai726	5,504.18 ± 381.68ab	102.09 ± 2.15d	981.71 ± 30.43ab	524.4 ± 19.06ab	841.97 ± 34.01cd	18.8 ± 9.63	7.88 ± 3.75	2.45 ± 0.02a	0.71 ± 0.02ab
Fu18	5,565.49 ± 386.16ab	169.31 ± 20.67e	1,483.39 ± 139.96c	639.11 ± 66.56bc	691.11 ± 26.83ab	25.21 ± 4.36	13.72 ± 4.33	2.61 ± 0.35a	0.59 ± 0.08ab
Fu22	5,833.89 ± 893.04ab	110.19 ± 15.3d	1,216.53 ± 28.6abc	564.43 ± 3.59ab	784.09 ± 12.64bc	13.1 ± 1.66	8.38 ± 4.8	6.91 ± 6.25b	0.78 ± 0.1b
Fu23	5,664.02 ± 141.55ab	179.21 ± 25.89e	1,133.23 ± 105.2abc	550.44 ± 63.83ab	728.77 ± 0.87abc	19.61 ± 7.24	7.74 ± 2.74	2.67 ± 0.03a	0.8 ± 0b
Tainong71	5,940.31 ± 50.38ab	161.54 ± 47.23e	1,242.43 ± 242.89abc	574.87 ± 82.21abc	753.97 ± 32.57abc	18.44 ± 1.07	10.36 ± 2.28	2.56 ± 0.29a	0.62 ± 0.03ab
Shulv1	6,843.2 ± 1,240.94b	37.33 ± 3.41a	1,117.76 ± 150.3abc	652.62 ± 88.1bc	952.52 ± 89.8d	14.71 ± 1.73	8.48 ± 2.89	2.7 ± 0.46a	0.74 ± 0.17b
Pushu53	5,168.17 ± 1,176.49a	82.83 ± 9.5bcd	1,189.01 ± 240.88abc	601 ± 100.12bc	731.69 ± 71.82abc	14.78 ± 2.79	8.75 ± 2.76	2.36 ± 1.03a	0.68 ± 0.15ab
Ningcai	5,952.33 ± 13.46ab	81 ± 17.7bcd	884.32 ± 167.69a	423.88 ± 20.97a	811.46 ± 126.79bc	17.64 ± 1.52	6.84 ± 1.93	2.77 ± 0.16a	0.68 ± 0.07ab

Cultivars	Vitamin E	Vitamin B_1_	Vitamin B_2_	Vitamin B_3_	Vitamin C	Folic acid^a^	β‐carotene	TPC	Antioxidant activity
Guangcai2	8.47 ± 0.15h	0.16 ± 0c	4.41 ± 0.03g	0.56 ± 0	16.4 ± 0.13d	56.41 ± 0.43fg	76.31 ± 4.23d	1.91 ± 0.24b	7.69 ± 0.13de
Gunagcai5	8.73 ± 0.28h	0.27 ± 0h	4.69 ± 0.03j	0.56 ± 0	35.5 ± 0.18f	54.35 ± 0.3cd	73.74 ± 3.68d	2.91 ± 0.42de	16.75 ± 0.51h
Ecai1	6.85 ± 0.2f	0.13 ± 0a	4.52 ± 0.01h	0.56 ± 0.01	146.45 ± 1.06k	53.45 ± 0.01ab	119.28 ± 2.57g	2.83 ± 0.28d	14.02 ± 1.12g
Ecai10	6.33 ± 0.23de	2.26 ± 0.01k	4.28 ± 0.03f	0.57 ± 0	44.54 ± 0.29g	56.46 ± 0.34fg	47.92 ± 4.02a	3.09 ± 0.15de	4.71 ± 0.29ab
Zhecai1	6.06 ± 0.06d	0.17 ± 0d	4.05 ± 0.01de	0.58 ± 0	18 ± 0.08e	52.99 ± 0.23a	105.14 ± 5.35f	1.82 ± 0.09b	7.08 ± 0.35cd
Zhecai726	6.23 ± 0.16d	0.12 ± 0a	4.05 ± 0de	0.57 ± 0	152.95 ± 0.92l	55.8 ± 0.95ef	82.95 ± 0.39e	4.16 ± 0.09f	3.94 ± 1.05a
Fu18	4.77 ± 0.08b	0.24 ± 0f	4.56 ± 0.01i	0.57 ± 0	12.42 ± 0.21b	56.27 ± 0.04fg	78.6 ± 0.74de	1.95 ± 0.15b	5.74 ± 0.57bc
Fu22	7.29 ± 0.18g	0.2 ± 0e	4.03 ± 0.01d	0.58 ± 0	75.37 ± 0.62h	56.84 ± 0.05gh	105.84 ± 0.24f	3.29 ± 0.04e	14.45 ± 1.12g
Fu23	5.37 ± 0.06c	0.32 ± 0i	4.09 ± 0.01e	0.56 ± 0	13.83 ± 0.23c	57.39 ± 0.07h	114.96 ± 0.38g	2.42 ± 0.02c	8.55 ± 0.33e
Tainong71	8.59 ± 0.39h	0.13 ± 0b	4.27 ± 0.01f	0.56 ± 0	143.1 ± 0.99j	56.19 ± 0.08fg	66.51 ± 1.07c	4.37 ± 0.09f	15.87 ± 0.27h
Shulv1	6.72 ± 0.17ef	0.43 ± 0j	3.8 ± 0.02b	0.58 ± 0.02	10.78 ± 0.13a	55.06 ± 0.22de	58.19 ± 3.85b	0.79 ± 0.05a	4.27 ± 0.16a
Pushu53	8.75 ± 0.03h	0.25 ± 0g	3.84 ± 0.01c	0.57 ± 0.01	125.9 ± 0.85i	54.15 ± 0.4bc	78.7 ± 1.7de	4.11 ± 0.15f	16.44 ± 0.73h
Ningcai	4.33 ± 0.07a	0.17 ± 0d	3.7 ± 0.02a	0.58 ± 0	12.88 ± 0.08bc	56.48 ± 0.22fg	117.79 ± 1.15g	1.77 ± 0.02b	11.76 ± 0.05f

Note

Data are means ± *SD* (*n* ≥ 2). Values within columns with different letters for minerals, vitamins, TPC or antioxidant activity are significantly different (*p* < .05).

Abbreviation: DW, dry weight.

^a^ Folic acid was expressed in μg/100 g DW.

The most abundant macroelement was K (average content of 6,065.63 mg/100 g DW), followed by Ca (average content of 1,289.57 mg/100 g DW), P (average content of 769.18 mg/100 g DW), Mg (average content of 628.03 mg/100 g DW), and Na (average content of 108.93 mg/100 g DW). K is important for the maintenance of fluid and electrolyte balance in body cells. Insufficient intake of K from the diet leads to hypokalemia, which contributes to life‐threatening conditions such as cardiac arrhythmias and acute respiratory failure. Mg is essential in nucleic acid synthesis. Low Mg levels have been associated with several diseases including asthma, diabetes, and osteoporosis.

Fe ranged from 11.93 (Fu22) to 41.02 (Guangcai2) mg/100 g DW, Mn ranged from 4.98 (Fu22) to 10.66 (Fu18) mg/100 g DW, Zn ranged from 2.53 (Guangcai5) to 11.33 (Fu22) mg/100 g DW, and Cu ranged from 0.54 (Guangcai5) to 0.91 (Ecai1) mg/100 g DW. The most abundant microelement was Fe (average content of 20.57 mg/100 g DW), followed by Mn (average content of 6.63 mg/100 g DW), Zn (average content of 3.39 mg/100 g DW), and Cu (average content of 0.72 mg/100 g DW). Even though heme iron from meat is more bioavailable than nonheme iron from sweet potato leaves, the intake of heme Fe/hemoglobin from red meat may increase the risk of colorectal cancer (Wang & Farid, 2015). Mn is related to the oxidative stress system and participates in glucose homeostasis and calcium transport. Zn is a component of several metallo‐enzymes. It is related to the metabolism of RNA and DNA, involved in gene expression, signal transduction, and so on. Cu is involved in the synthesis of collagen and various enzymatic reactions.

### 3.3 Vitamin content

The vitamin content of sweet potato leaves from different cultivars is presented in Table 2. VB_1_ can maintain the normal functions of circulation, digestion, nerve, and muscle, and adjust the function of gastrointestinal tract. VB_1_ content ranged from 0.12 (Zhecai726) to 2.26 (Ecai10) mg/100 g DW. VB_2_ is a component of many important coenzymes in the body. These enzymes can transfer hydrogen in the process of substance metabolism, promote growth and development, and protect the health of eyes and skin.VB_2_ content ranged from 3.7 (Ningcai) to 4.69 (Guangcai5) mg/100 g DW. VB_3_ can be converted into nicotinamide and participate in lipid metabolism, oxidation of tissue respiration, and anaerobic decomposition of carbohydrates.VB_3_ content has no significant difference among different cultivars. VC is important in wound healing and in the prevention of scurvy, and it is an antioxidant that minimizes oxidative stress (Lee et al., 2013). VC content ranged from 10.78 (Shulv1) to 152.95 (Zhecai726) mg/100 g DW. VE takes charge of normal reproductive capacity and muscle metabolism, and keep the integrity of the central nervous and vascular system. VE content ranged from 4.33 to 8.75 mg/100 g DW. The function of folic acid is anti‐anemia and defends the normal growth of cells and the function of the immune system. The folic acid content ranged from 52.99 to 56.84 μg/100 g DW and β‐carotene content ranged from 47.92 to 119.23 mg/100 g DW. Additionally, β‐carotene is a precursor to the synthesis of VA and helps to protect the body from free radicals.

### 3.4 Amino acid composition and evaluation

The AAS information of 13 sweet potato leaves is shown in Table 3. The first limiting amino acid of all samples was methionine + cysteine, which is the same results with the study of seaweeds from the Magellan Straits (Astorga‐españa, Rodríguez‐galdón, Rodríguez‐rodríguez, & Díaz‐romero, 2016). The total amino acids (TAA) include essential and semi‐essential amino acid (EAA) and nonessential amino acid (NEAA). Shulv1 exhibited the highest TAA content of 19.23 g/100 g DW. The same was observed for EAA content at 7.26 g/100 g DW. The nutrition value of sweet potato leave protein was further evaluated by the AAS. Ningcai had the highest AAS of 32.58. This indicated that the amino acid composition of Ningcai was relatively balanced. Thus, Ningcai may possess a good protein quantity.

**TABLE 3:** Amino acid composition of leaves from 13 sweet potato cultivars (g/100 g DW)

Amino acids	Guangcai2	Guangcai5	Ecai1	Ecai10	Zhecai1	Zhecai726	Fu18	Fu22	Fu23	Tainong71	Shulv1	Pushu53	Ningcai
EAA													
Threonine	0.80 ± 0.00	0.79 ± 0.02	0.80 ± 0.05	0.78 ± 0.02	0.79 ± 0.04	0.80 ± 0.02	0.78 ± 0.02	0.77 ± 0.04	0.74 ± 0.01	0.81 ± 0.02	0.85 ± 0.01	0.72 ± 0.01	0.76 ± 0.03
Cysteine	0.03 ± 0.00	0.03 ± 0.01	0.03 ± 0.00	0.04 ± 0.01	0.04 ± 0.00	0.03 ± 0.01	0.03 ± 0.00	0.02 ± 0.00	0.03 ± 0.00	0.02 ± 0.00	0.03 ± 0.01	0.03 ± 0.01	0.03 ± 0.00
Valine	0.99 ± 0.02	0.95 ± 0.04	0.98 ± 0.05	0.99 ± 0.02	0.98 ± 0.04	0.98 ± 0.04	0.97 ± 0.02	0.94 ± 0.05	0.92 ± 0.02	0.99 ± 0.02	1.06 ± 0.00	0.88 ± 0.02	0.92 ± 0.05
Methionine	0.14 ± 0.02	0.14 ± 0.01	0.15 ± 0.00	0.07 ± 0.02	0.10 ± 0.02	0.14 ± 0.02	0.12 ± 0.01	0.12 ± 0.02	0.11 ± 0.00	0.12 ± 0.01	0.13 ± 0.01	0.11 ± 0.00	0.14 ± 0.04
Isoleucine	0.77 ± 0.00	0.75 ± 0.03	0.79 ± 0.04	0.78 ± 0.02	0.77 ± 0.04	0.76 ± 0.03	0.77 ± 0.02	0.75 ± 0.03	0.73 ± 0.01	0.79 ± 0.01	0.83 ± 0.00	0.69 ± 0.01	0.73 ± 0.04
Leucine	1.42 ± 0.00	1.38 ± 0.05	1.46 ± 0.08	1.46 ± 0.03	1.42 ± 0.07	1.41 ± 0.05	1.44 ± 0.03	1.4 ± 0.07	1.35 ± 0.03	1.47 ± 0.04	1.55 ± 0.01	1.29 ± 0.02	1.38 ± 0.06
Tyrosine	0.46 ± 0.01	0.49 ± 0.00	0.52 ± 0.03	0.35 ± 0.01	0.42 ± 0.04	0.47 ± 0.02	0.44 ± 0.03	0.48 ± 0.02	0.43 ± 0.02	0.48 ± 0.01	0.47 ± 0.01	0.46 ± 0.02	0.45 ± 0.02
Phenylalanine	0.99 ± 0.01	0.97 ± 0.02	1.01 ± 0.05	1.03 ± 0.03	1.00 ± 0.05	1.00 ± 0.03	1.01 ± 0.02	0.97 ± 0.03	0.94 ± 0.04	1.03 ± 0.03	1.09 ± 0.01	0.9 ± 0.04	0.93 ± 0.04
Lysine	1.18 ± 0.01	1.18 ± 0.03	1.19 ± 0.06	1.13 ± 0.04	1.12 ± 0.06	1.16 ± 0.03	1.15 ± 0.03	1.18 ± 0.05	1.08 ± 0.02	1.19 ± 0.03	1.27 ± 0.01	1.11 ± 0.03	1.13 ± 0.02
NEAA													
Asparagine	2.84 ± 0.02	2.26 ± 0.07	2.19 ± 0.13	2.62 ± 0.10	2.38 ± 0.14	2.50 ± 0.07	2.33 ± 0.04	2.2 ± 0.13	2.47 ± 0.04	2.29 ± 0.03	2.69 ± 0.01	1.99 ± 0.05	2.10 ± 0.09
Serine	0.79 ± 0.00	0.78 ± 0.02	0.75 ± 0.05	0.77 ± 0.02	0.80 ± 0.04	0.78 ± 0.01	0.74 ± 0.01	0.73 ± 0.03	0.72 ± 0.01	0.76 ± 0.02	0.81 ± 0.01	0.71 ± 0.01	0.73 ± 0.03
Glutamic acid	2.58 ± 0.00	2.32 ± 0.08	2.64 ± 0.16	2.53 ± 0.08	2.34 ± 0.12	2.46 ± 0.07	2.37 ± 0.05	2.40 ± 0.11	2.56 ± 0.05	2.52 ± 0.06	2.68 ± 0.02	2.25 ± 0.04	2.45 ± 0.11
Glycine	0.92 ± 0.00	0.89 ± 0.04	0.95 ± 0.06	0.96 ± 0.02	0.97 ± 0.05	0.95 ± 0.04	0.93 ± 0.02	0.91 ± 0.04	0.88 ± 0.02	0.95 ± 0.03	1.01 ± 0.01	0.85 ± 0.01	0.89 ± 0.04
Alanine	1.00 ± 0.01	0.96 ± 0.04	1.00 ± 0.06	1.00 ± 0.02	1.01 ± 0.06	0.99 ± 0.04	0.99 ± 0.02	0.95 ± 0.05	0.94 ± 0.02	1.00 ± 0.03	1.07 ± 0.01	0.88 ± 0.01	0.98 ± 0.03
Histidine	0.41 ± 0.00	0.39 ± 0.01	0.41 ± 0.02	0.41 ± 0.02	0.39 ± 0.01	0.39 ± 0.02	0.39 ± 0.01	0.40 ± 0.02	0.38 ± 0.00	0.40 ± 0.01	0.42 ± 0.00	0.38 ± 0.02	0.41 ± 0.00
Arginine	0.99 ± 0.01	1.00 ± 0.03	1.02 ± 0.05	1.02 ± 0.02	0.97 ± 0.05	1.01 ± 0.04	1.00 ± 0.03	0.95 ± 0.05	0.93 ± 0.02	1.03 ± 0.03	1.10 ± 0.01	1.02 ± 0.02	0.99 ± 0.01
Proline	2.06 ± 0.00	1.85 ± 0.01	2.18 ± 0.09	2.04 ± 0.02	1.90 ± 0.13	2.00 ± 0.06	1.93 ± 0.04	1.91 ± 0.06	2.09 ± 0.03	2.08 ± 0.09	2.18 ± 0.04	1.83 ± 0.00	2.06 ± 0.00
EAA	6.73 ± 0.01	6.65 ± 0.22	6.89 ± 0.37	6.58 ± 0.15	6.60 ± 0.35	6.72 ± 0.23	6.67 ± 0.16	6.6 ± 0.30	6.29 ± 0.15	6.87 ± 0.18	7.26 ± 0.02	6.15 ± 0.15	6.45 ± 0.30
TAA	18.35 ± 0.02	17.11 ± 0.54	18.06 ± 0.99	17.96 ± 0.46	17.40 ± 0.96	17.82 ± 0.58	17.38 ± 0.38	17.08 ± 0.80	17.29 ± 0.33	17.93 ± 0.48	19.23 ± 0.11	16.08 ± 0.31	16.88 ± 0.75
EAA/TAA	0.37	0.39	0.38	0.38	0.38	0.38	0.39	0.37	0.39	0.38	0.38	0.37	0.39
AAS^a^	30.68	32.08	31.98	19.03	27.08	31.30	26.09	28.57	25.28	25.85	28.62	27.20	32.58

Note

Data are means ± *SD* (*n* ≥ 2).

Abbreviations: EAA, essential and semi‐essential amino acid; NEAA, nonessential amino acid; TAA, total amino acid content.

^a^ The AAS results were calculated according to the WHO/FAO/UNO (2007) adult essential amino acid requirement pattern.

### 3.5 Comprehensive nutritional value

**FIGURE 1:**
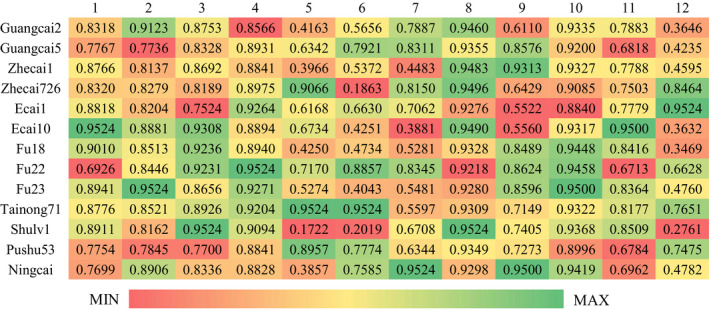
The weighted gray relational grades (WGRG) heat map of leaves from 13 sweet potato cultivars

The content of one specific nutrient is not indicative of overall quality. Therefore, it is important to perform a comprehensive nutritional analysis. In this study, gray relational analysis was performed to assess the comprehensive nutritional value of 13 different cultivars (Table S1). The results revealed that varieties significantly affected nutritional values. The heat map (Figure 1) reflected the influence of every factor on the final results and explained the differences among the results. Tainong71 possessed the largest number of green parts, which represented the closeness to the ideal cultivar. Meanwhile, the heat maps for Zhecai1 showed more red and yellow parts, indicating that they had the lowest rankings. The rankings of all of the cultivars are shown in Table S2. In decreasing order of gray relational grade values was Tainong71 (0.8492) > Fu22 (0.8217) > Ningcai (0.8047) > Fu23 (0.8044) > Ecai10 (0.7903) > Zhecai726 (0.7880) > Ecai1 (0.7854) > Fu18 (0.7800) > Pushu53 (0.7787) > Guangcai5 (0.7786) > Shulv1 (0.7658) > Guangcai2 (0.7625) > Zhecai1 (0.7606). The results indicate that Tainong71 is the most approach to the ideal variety, followed by Fu22 and Ningcai. GRA has been recognized as comprehensive and less limited by factors, reasonable and natural, and can be processed by computer technology. It avoids the disadvantage that the previous evaluation only considers crude protein, crude fat, and crude fiber while ignoring other factors, so the evaluation results are more objective and accurate.

### 3.6 Sunscreen activity

The Sun Protection Factor (SPF) value with different concentration of sweet potato ethanol extract was shown in Table 4. Different concentrations of ascorbic acid were taken as positive control. The SPF value was increased gradually with the increase of concentration. The variety with highest SPF is Pushu53, followed by Guangcai5 whereas the lowest was Shulv1. There were significant differences (*p* < .05) in SPF among different sweet potato cultivars, which was probably attributed to differences in genotype and other composition in sweet potato leaves. The maximum SPF of the sweet potato leaf ethanol extract we measured was 24.65 (Pushu53), while it was observed that the SPF values of topical applications were validated up to 30 SPF (Prakash et al., 2015).

**TABLE 4:** SPF of sweet potato leaf extract with different concentrations (μg/ml)

Cultivars	10	100	200	300	400	500	600	700
Guangcai2	0.43 ± 0.001d	2.2 ± 0.001g	2.52 ± 0h	3.63 ± 0.003h	4.83 ± 0.018i	6.14 ± 0.001g	6.72 ± 0.003h	8.87 ± 0.002h
Guangcai5	0.5 ± 0.003h	3.06 ± 0.006l	4.26 ± 0.038l	6.26 ± 0.001m	8.37 ± 0.001m	10.71 ± 0.122l	13.26 ± 0.001l	22.47 ± 0.004m
Ecai1	0.44 ± 0.001d	2.55 ± 0.002b	3.28 ± 0.01c	4.65 ± 0.001d	6.21 ± 0.004d	9.29 ± 0.006d	10 ± 0.006d	13.06 ± 0.003d
Ecai10	0.35 ± 0.007b	1.78 ± 0.002d	1.39 ± 0.007d	1.95 ± 0.006e	2.51 ± 0.002e	3.38 ± 0.007d	3.43 ± 0.002e	4.38 ± 0.007e
Zhecai1	0.41 ± 0.004c	1.95 ± 0.006g	1.94 ± 0.008k	2.85 ± 0.002l	3.61 ± 0.002l	4.82 ± 0.003k	5.21 ± 0.003j	6.6 ± 0.004l
Zhecai726	0.36 ± 0.003b	1.76 ± 0.004f	1.43 ± 0.002f	2.06 ± 0.002i	2.61 ± 0.006h	3.39 ± 0.002h	3.61 ± 0.005h	4.59 ± 0.001i
Fu18	0.45 ± 0.003e	1.85 ± 0.004j	1.88 ± 0.003j	2.67 ± 0.018k	3.3 ± 0.006k	4.43 ± 0.005j	4.83 ± 0.002k	6.3 ± 0.109k
Fu22	0.49 ± 0.003g	2.55 ± 0.002c	3.54 ± 0.002b	5.09 ± 0.002c	6.39 ± 0.002c	10.33 ± 0.007c	9.83 ± 0.125c	13.86 ± 0.007c
Fu23	0.44 ± 0.007d	2.05 ± 0.004e	2.31 ± 0.003e	3.67 ± 0.006f	4.71 ± 0.002f	6.52 ± 0.007e	6.76 ± 0.003f	8.99 ± 0.002f
Tainong71	0.46 ± 0.002f	2.27 ± 0.001h	2.42 ± 0.003g	3.54 ± 0.002g	4.38 ± 0.001g	5.86 ± 0.005f	6.33 ± 0.02g	8.31 ± 0g
Shulv1	0.34 ± 0.007a	1.58 ± 0.003a	1.37 ± 0.007b	1.92 ± 0.006b	2.46 ± 0.002b	3.15 ± 0.003b	3.24 ± 0.007b	4.26 ± 0.006b
Pushu53	0.5 ± 0.002h	2.96 ± 0.003k	4.44 ± 0.006m	6.55 ± 0.007n	8.61 ± 0.002n	11.54 ± 0.191m	14.06 ± 0.002m	24.65 ± 0.006n
Ningcai	0.45 ± 0.001f	2.33 ± 0.007i	2.57 ± 0.003i	3.91 ± 0.002j	5.29 ± 0.002j	7.08 ± 0.002i	7.39 ± 0.003i	9.95 ± 0.005j
Ascorbic acid	0.44 ± 0.002e	1.53 ± 0.004a	0.73 ± 0.039a	0.7 ± 0.005a	0.73 ± 0.002a	0.98 ± 0.007a	0.86 ± 0.007a	0.93 ± 0.002a

Note

Data are means ± *SD* (*n* ≥ 2). Values within columns with different letters are significantly different (*p* < .05).

SPF is a standard for quantitatively measuring the effectiveness of sunscreen which is faster and simpler than human body method. At present, chemical sunscreen agents such as methoxy cinnamate ethyl hexyl ester, butyl methoxy dibenzoyl methane are commonly used in cosmetics. However, these sunscreen agents may induce photosensitization (Collaris & Frank, 2008). Therefore, sweet potato leaves have potential to become urgently needed natural plant sunscreen agents.

### 3.7 Antioxidant activity

Antioxidant activity was determined by the FRAP method, and the results are shown in Table 2. Pushu53 had the highest antioxidant activity (16.44 ± 0.73 g TE/g DW), whereas Zhecai726 had the lowest antioxidant activity (3.94 ± 1.05 g TE/g DW). The antioxidant usually considered to be attributed to different TPC, polyphenol types, and nutrient composition, which maybe has synergistic or antagonistic effects on the antioxidant activity.

**FIGURE 2:**
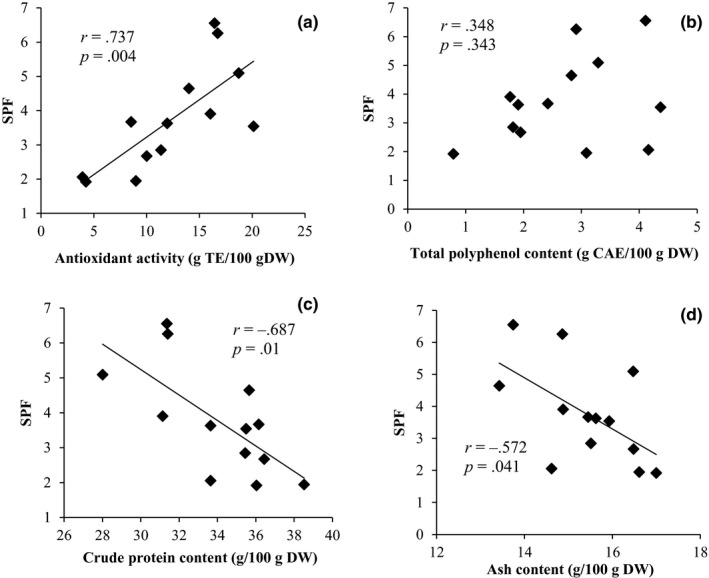
Correlation coefficient between sun protection factor (SPF) and antioxidant activity (a), total polyphenol content (b), crude protein content (c), and ash content (d) at 300 μg/ml of sweet potato leaf extract

The correlations between SPF at 300 μg/ml and antioxidant activity, TPC, crude protein content, and crude fiber content are shown in Figure 2. The correlation coefficient between antioxidant activity and SPF of sweet potato leaves (*r* = .737; *p* = .004) was highest. Followed by is the correlation coefficient between TPC and SPF (*r* = .348; *p* = .243). There were negative correlation coefficients between SPF and crude protein (*r* = −.687, *p* = .010); then, the correlation coefficient between ash content and SPF is (*r* = −.572; *p* = .041). UV radiation can stimulate the activity of oxidase, damage the role of antioxidants, and lead to oxidative stress (Gęgotek, Ambrożewicz, Jastrząb, Jarocka‐Karpowicz, & Skrzydlewska, 2019), so the varieties with strong sunscreen activity will also be accompanied by high antioxidant capacity. It has also been reported that there is a negative correlation between antioxidant capacity and protein content (Liu et al., 2017), which may contribute to the negative correlation between SPF and protein content. Therefore, antioxidant activity is considered to be the most important in resisting ultraviolet in sweet potato leaves. Because of their diversity and wide distribution, so may be many natural antioxidants exist in sweet potato leaves, which play significant roles in the organoleptic and nutritional qualities of fruits and vegetables.

## 4 CONCLUSION

There were significant differences in proximate composition among the sweet potato cultivars. GRA reveals that the best variety of comprehensive nutritional quality is Tainong71, followed by Fu22. Sweet potato leaves have good sunscreen activity. Antioxidant activity is the most important factor associated with SPF. In conclusion, sweet potato leaves which contain abundant nutrients and bioactive compounds should be consumed as leafy vegetables in an attempt to supplement nutrition and have big potential to become a new natural plant sunscreen agent.

## CONFLICT OF INTEREST

The authors have no conflicts of interest to declare.

## Supporting information

File S1
