# Supplementary material for: Valorization of the green waste parts from sweet potato (Impoea batatas L.): Nutritional, phytochemical composition, and bioactivity evaluation
Source: Food Sci Nutr. 2020 Jul 14;8(8):4086–97. doi: 10.1002/fsn3.1675 (PMC7455964; doi:10.1002/fsn3.1675)
Supplement: Supplementary file 1 — File S1 [file FSN3-8-4086-s001.docx]

**Supplementary table captions**

Table S1. Computed result of leaves from 13 sweet potato cultivars with dimensionless method

Table S2. Grey relational degree and rank of leaves from 13 sweet potato cultivars

| Cultivars | Standard | Guangcai2 | Guangcai5 | Ecai1 | Ecai10 | Zhecai1 | Zhecai726 | Fu18 | Fu22 | Fu23 | Tainong71 | Shulv1 | Pushu53 | Ningcai1 |
| --- | --- | --- | --- | --- | --- | --- | --- | --- | --- | --- | --- | --- | --- | --- |
| Moisture | 1 | 0.9460 | 0.9355 | 0.9276 | 0.9490 | 0.9483 | 0.9496 | 0.9328 | 0.9218 | 0.9280 | 0.9309 | 0.9524 | 0.9349 | 0.9298 |
| Crude fat | 1 | 0.6110 | 0.8576 | 0.5522 | 0.5560 | 0.9313 | 0.6429 | 0.8489 | 0.8624 | 0.8596 | 0.7149 | 0.7405 | 0.7273 | 0.9500 |
| Crude protein | 1 | 0.8318 | 0.7767 | 0.8818 | 0.9524 | 0.8766 | 0.8320 | 0.9010 | 0.6926 | 0.8941 | 0.8776 | 0.8911 | 0.7754 | 0.7699 |
| Crude fiber | 1 | 0.9123 | 0.7736 | 0.8204 | 0.8881 | 0.8137 | 0.8279 | 0.8513 | 0.8446 | 0.9524 | 0.8521 | 0.8162 | 0.7845 | 0.8906 |
| Carbohydrate | 1 | 0.7883 | 0.6818 | 0.7779 | 0.9500 | 0.7788 | 0.7503 | 0.8416 | 0.6713 | 0.8364 | 0.8177 | 0.8509 | 0.6784 | 0.6962 |
| Gross energy | 1 | 0.9335 | 0.9200 | 0.8840 | 0.9317 | 0.9327 | 0.9085 | 0.9448 | 0.9458 | 0.9500 | 0.9322 | 0.9368 | 0.8996 | 0.9419 |
| Ash | 1 | 0.8753 | 0.8328 | 0.7524 | 0.9308 | 0.8692 | 0.8189 | 0.9236 | 0.9231 | 0.8656 | 0.8926 | 0.9524 | 0.7700 | 0.8336 |
| Dietary fiber | 1 | 0.8566 | 0.8931 | 0.9264 | 0.8894 | 0.8841 | 0.8975 | 0.8940 | 0.9524 | 0.9271 | 0.9204 | 0.9094 | 0.8841 | 0.8828 |
| Amino acid score | 1 | 0.7887 | 0.8311 | 0.7062 | 0.3881 | 0.4483 | 0.8150 | 0.5281 | 0.8345 | 0.5481 | 0.5597 | 0.6708 | 0.6344 | 0.9524 |
| Total polyphenol content | 1 | 0.4163 | 0.6342 | 0.6168 | 0.6734 | 0.3966 | 0.9066 | 0.4250 | 0.7170 | 0.5274 | 0.9524 | 0.1722 | 0.8957 | 0.3857 |
| Antioxidant activity | 1 | 0.5656 | 0.7921 | 0.6630 | 0.4251 | 0.5372 | 0.1863 | 0.4734 | 0.8857 | 0.4043 | 0.9524 | 0.2019 | 0.7774 | 0.7585 |
| Total Vitamin* | 1 | 0.3646 | 0.4235 | 0.4595 | 0.8464 | 0.9524 | 0.3632 | 0.3469 | 0.6628 | 0.4760 | 0.7651 | 0.2761 | 0.7475 | 0.4782 |

Table S1

*Total vitamin means the sum of the Vitamin B_1_, Vitamin B_2_, Vitamin B_3_, Vitamin C, Vitamin E, folic acid and β-carotene.

Table S2

| Cultivars | Grey relation degree | Rank |
| --- | --- | --- |
| Guangcai2 | 0.7625^ab^ | 12 |
| Guangcai5 | 0.7786^bcd^ | 10 |
| Zhecai1 | 0.7606^a^ | 13 |
| Zhecai726 | 0.7880^de^ | 6 |
| Ecai1 | 0.7854^d^ | 7 |
| Ecai10 | 0.7903^de^ | 5 |
| Fu18 | 0.7800^cd^ | 8 |
| Fu22 | 0.8217^f^ | 2 |
| Fu23 | 0.8044^e^ | 4 |
| Tainong71 | 0.8492^g^ | 1 |
| Shulv1 | 0.7658^abc^ | 11 |
| Pushu53 | 0.7787^bcd^ | 9 |
| Ningcai1 | 0.8047^e^ | 3 |

Note: Values followed by different superscript letters in the column indicate significant differences (p<0.05)
